# Using an intervention mapping approach to develop prevention and rehabilitation strategies for musculoskeletal pain among surgeons

**DOI:** 10.1186/s12889-019-6625-4

**Published:** 2019-03-18

**Authors:** Tina Dalager, Anne Højmark, Pernille Tine Jensen, Karen Søgaard, Lotte Nygaard Andersen

**Affiliations:** 1 Department of Sports Science and Clinical Biomechanics, University of Southern Denmark, Campusvej 55, 5230 Odense M, Denmark; 2 Clinical Institute, University of Southern Denmark and Department of Gynaecology and Obstetrics, Odense University Hospital, Odense, Denmark; 3 Clinical Institute, University of Southern Denmark and Department of Occupational and Environmental Medicine, Odense University Hospital, Odense, Denmark

**Keywords:** Needs assessment, Logic model, Work-related pain, Workplace, Micro-breaks, Physical exercise training, Ergonomics, Practical strategies

## Abstract

**Background:**

The aim of the present study was to describe the development of strategies to prevent and rehabilitate musculoskeletal pain among surgeons. Musculoskeletal pain affects surgeons’ life, and evidence on interventions for effective prevention and rehabilitation is lacking for this occupational group.

**Methods:**

An Intervention Mapping approach was used to develop intervention strategies specifically tailored to surgeons. This approach entailed conducting a systematic scoping literature search and semi-structured interviews with six surgeons.

**Results:**

The first step was to develop a logic model of the problem of musculoskeletal pain among surgeons. Step two was to formulate health-enhancing outcomes and performance objectives for the intervention, while in step three theory-based methods and practical strategies for the intervention were identified.

**Conclusion:**

The present Intervention Mapping study demonstrated that musculoskeletal pain among surgeons is a complex area that needs attention. Our findings highlight a need for individual behavioural changes as well as organisational, attitudinal, and management changes.

## Background

A substantial amount of literature emphasises that surgeons are at high risk of developing work-related musculoskeletal pain [1–6]. Work-related musculoskeletal pain is a growing problem and accounts for approx. 40% of all occupational diseases in Europe [7]. The presence of musculoskeletal pain has been associated with reduced quality of life for the individual, reduced productivity and increased sickness absence at the workplace [7, 8]. The annual cost worldwide attributable to occupational pain has been estimated to be $560 to $635 billions [9], and in Europe it constitutes around 1.5 to 3% of the European gross domestic product [10]. Work-related musculoskeletal pain has, therefore, a significant health and socio-economic impact.

The operation theatre is a complex environment, and technological developments have been evaluated primarily from a patient perspective with little or no consideration of the impact on the surgeon’s working conditions [11, 12]. Repetitive motions and cumulative awkward postures are an everyday occurrence for surgeons. Studies report that surgeons lack the knowledge on how to adapt to less physically demanding work postures and often disregard their own comfort [13–17]. Until now, reducing surgeons’ pain has been attempted by focusing on reducing the physical workload by implementing new ergonomic equipment in the operating theatre, changing work posture, decreasing overall caseload, or implementing micro-breaks during surgery [18–20]. Only the latter has resulted in lower pain scores [21].

To our knowledge, no randomised controlled trials have evaluated interventions regarding preventive or rehabilitating strategies of musculoskeletal pain in surgeons. This is pertinent for this highly specialised group, who perform a variety of tasks and who often work in long shifts. To meet the specific needs in this group of workers and obtain good compliance, an intervention must be designed based on a participatory approach [22]. Involvement of the surgeons in the process is essential because it ensures relevance and ownership of risk identification, solution development, and implementation of change. To structure this process and ensure the development and implementation of a feasible and effective intervention, the concept of Intervention Mapping can be useful.

Intervention Mapping is a problem-driven and a theory-driven protocol, that includes knowledge obtained from the literature and involvement of key stakeholders [23]. It encompasses six key steps: 1) a needs assessment to identify the problem, 2) the identification of outcomes and change objectives, 3) the selection of theory-based methods and practical strategies, 4) the development of an intervention plan, 5) generation of adoption and implementation plan, and 6) the generation of an evaluation plan. Intervention Mapping is well expounded in the health promotion literature and has proven useful in other occupational settings for different outcomes [24–27].

The present study focusses on the first three steps in the overall framework of Intervention Mapping. The aim was to develop feasible strategies that may potentially prevent and rehabilitate musculoskeletal pain among surgeons by using Intervention Mapping.

## Methods

### Intervention mapping

**Fig. 1:**
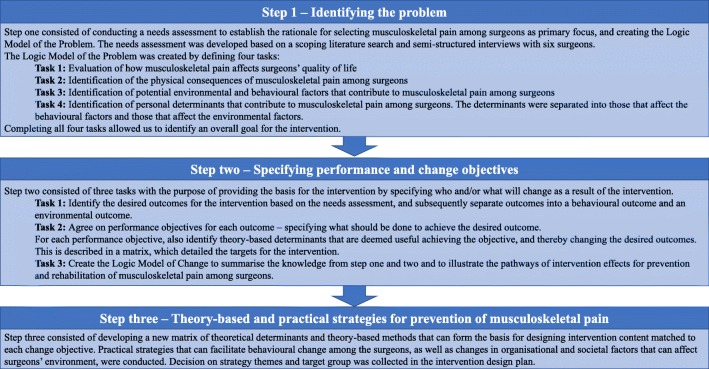
Illustration of step 1–3 in Intervention Mapping

An Intervention Mapping protocol was used to ensure the intervention that was developed was grounded in theory. The utility of basing interventions upon sound theoretical frameworks is that theory can help to identify effective methods for behavioural change, as well as explain and predict the process of change [23]. This study applied the first three steps in Intervention Mapping. Overall, step one was to define the problem, step two was to specify intervention outcomes and expected changes, and step three was to design a feasible and effective intervention with strategies that can alleviate problems that were identified in step one. The three different steps encompassed additionally specific tasks before moving to the next step [28, 29]. Figure 1 details the specific tasks in each step.

#### Literature search

A systematic literature search with a scoping approach was performed as part of the Intervention Mapping methodology [30]. In accordance with the scoping approach the systematic literature search was conducted in order to obtain in depth knowledge of any potential problems and challenges surgeons may present with. The knowledge was used to compose the semi-structured interview guide. The databases PubMed, Embase and CINAHL were searched until 17 April 2017 using the following search strategy adjusted to fit each database criteria: (surgeon*) AND (occupational exposure OR work-related exposure OR job-exposure OR job demands OR workload OR work-related) AND (musculoskeletal disorders OR musculoskeletal diseases OR musculoskeletal pain OR occupational diseases OR work-related diseases). Articles were selected based on 1) title, 2) abstract, and 3) full text. The systematic literature search was conducted by author AH and continuously discussed with author LNA and author TD.

#### Semi-structured interviews

Semi-structured interviews were conducted face-to-face with surgeons to understand the surgeon’s type of job, its influence on health, and their experiences with health initiatives. Based on the literature search, a semi-structured interview guide was designed. It evaluated the issues of the surgeons’ workplace, their experiences with pain, approach to their health, and their experiences with health initiatives as well as their experiences of challenges concerning such health initiatives. Interview questions were formulated in everyday language and were preceded by descriptive questions to enhance confidence and familiarity between the interviewed surgeon and the interviewer. The semi-structured interview guide was discussed between co-authors before conducting the interviews. Only the interviewer (author AH) and the participant were present during the interview.

Interviews were conducted in Danish, audio-recorded and transcribed verbatim by author AH. Participants were subsequently asked to confirm the accuracy of the content. The quotations used in the present paper were translated from Danish to English by a professional translator.

The analyses were based on systematic text condensation, which is a descriptive and explorative method used for thematic cross-case analysis [31]. It is a four-step process derived from theories of phenomenology and supports the surgeons’ experiences being expressed in their own words.

Recruitment for the interview was done by author TD and an established contact person at two medical wards (Gastrointestinal and Gynaecology and Obstetrics) at two distinct hospitals. Recruitment was based on earlier contact and therefore convenience sampling was used. Surgeons interested in participation were sent written information and ensured complete anonymity. All interested surgeons agreed to participate.

## Results

### Step 1: Identifying the problem

#### Needs assessment

Results from the literature search and semi-structured interviews will be presented to enable a detailed description of determinants that influence musculoskeletal pain among surgeons. It was on the basis of these results that the logic model of the problem was developed, and the overall goal was formulated.

#### Literature search

The systematic literature search initially identified 179 articles (Pubmed: 71, Embase: 95, CINAHL: 13). Based on the review procedure 14 articles were considered relevant. Among the relevant articles was one systematic review [32] including 35 studies about the prevalence of musculoskeletal pain among surgeons performing minimally invasive surgery. All types of study design were searched. Except for the systematic review only studies with quantitative methodology were identified. The literature search supported that work-related musculoskeletal pain is a common and documented problem among surgeons [5, 33, 34]. Surgeons have long workdays and a physically demanding job. They are exposed to cumulative awkward postures, repetitive movement, and strenuous exertion during the performance of surgery, which may lead to muscular strain and ultimately the development of musculoskeletal pain [6, 18]. A systematic literature review from 2017 included 35 studies and found a prevalence of musculoskeletal pain among surgeons varying between 73 to 88% [32]. A prevalence well above that in the general working population [7]. The most prevalent painful body regions were the neck and the lower back [14, 18, 20, 34]. Studies report that surgeons believe their surgical performance is affected by pain [5, 20], and that surgeons do not pay attention to their own health during surgery [12, 13]. In a study by Park et al., 40% of the surgeons reported to disregard their physical complaints [15]. Another study showed that 36% of the surgeons reported to work in spite of pain, as they considered pain to be part of the job [14]. In addition, many surgeons reported to be unaware of physical ergonomic guidelines relevant to the operating theatre [16, 17].

#### Semi-structured interviews

Six surgeons from two Danish hospitals volunteered to participate in the present study. They were interviewed at their workplace in an undisturbed setting and with a duration of 52–78 min. All the surgeons were experienced and had worked as surgeons for six to 20 years. Weekly working hours were reported to be between 45 and 60 h. Musculoskeletal pain was present for four out of six surgeons. Characteristics for the six surgeons are shown in Table 1 (study participants’ names are pseudonyms).

**Table 1:** Participants’ characteristics

Participant	Gender	Age range (years)	Surgical specialty	Years in MIS	Presence of musculoskeletal pain	Body region with pain
Christian	Male	50–59	Gastrointestinal	+ 20	Yes	Neck and lower back
Martin	Male	40–49	Gastrointestinal	11–15	Yes	Neck, shoulder, hands, lower back and knees
David	Male	40–49	Gastrointestinal	6–10	No	
Andreas	Male	40–49	Gynaecology and Obstetrics	11–15	Yes	Neck and lower back
Pia	Female	50–59	Gynaecology and Obstetrics	16–20	No	
Susanne	Female	50–59	Gynaecology and Obstetrics	6–10	Yes	Neck and lower back

All participants’ names are pseudonyms

We applied systematic text condensation to identify themes in the interviews [31]. The analysis identified four main themes derived from the data: (1) Experienced challenges; (2) Surgeons do not complain; (3) Taking responsibility; (4) How to handle it. Additionally, three subthemes were identified under the fourth theme: (a) work-related focus on physical ergonomics, (b) Micro-breaks and physical activity and (c) Experiences with health promotion initiatives. In the following, the results from the interviews are presented.

##### Experienced challenges

A male surgeon described it as a catastrophe if he cannot operate because it is the primary function in his job. He has concerns about his surgical career, because of his musculoskeletal pain:

*“I suffer from chronic neck pain ( … ) Can that be recognised as a work-related injury? It is a work-related injury, I think. It’s not like I have other things that affect my neck in that way ( … ) I don’t know about the future. You wonder what it will mean when you get older, after 15- or 20-years work, if I already have pain in my neck now.” (Andreas)*

Lengthy operation times and strenuous work postures for many hours, are a particular burden for the surgeons. Several of the surgeons had witness that colleagues had been forced to stop their career as a surgeon or to change the work they perform:

*“One of my colleagues who is a professor, has pretty much stopped operating. His back simply gave him so much pain that he was beside himself, so now he is mostly professor and manager, but it’s a waste of a surgeon, for he is extremely able.” (Susanne)*

##### Surgeons do not complain

Among surgeons, there is no tradition for talking about their work-related musculoskeletal pain. A surgeon described his experience with handling a piriformis syndrome at work:

*“Surgeons are not the type of people who go and complain ( … ) no one has the least idea that I have a piriformis syndrome. I’ve never mentioned it, or maybe there was a day if I could hardly walk. But it’s not something you talk about, though everyone is affected by work postures.” (Martin)*

The surgeons did not share concerns with colleagues about their work-related musculoskeletal pain. It is as if there is a mutual acceptance about musculoskeletal pain being a natural consequence of their work.

*“There’s no doubt that, if you’re going to be in a surgical environment, you have to have a particular temperament ( … ) those who don’t have it, have a hard time ( … ) it’s like this: I’m damned well not going to be the one who can’t cope with it.” (Susanne)*

From a long-term perspective, the surgeons are conscious of the fact that musculoskeletal pain may challenge their chance of maintaining their job.

##### Taking responsibility

During an operation, surgeons are very clear about their priorities and feel a great responsibility for the patient’s care. Despite musculoskeletal pain related to their work posture and duration of operation, one surgeon describe how she would never interrupt or cease an operation:

*“Once I have said that I will operate on her, I can’t simply walk away from her, I mean, I can’t let the patient lie there ( … ). It’s a human being, you can’t let them lie there, she’s been put to sleep, I mean her stomach’s open?” (Susanne)*

Feelings of responsibility for patients were expressed by several of the surgeons interviewed. This prohibited the surgeon from stopping or taking a break. On the other hand, one surgeon mentioned that she was also conscious about the responsibility to herself. She was obliged to consider whether she should call another surgeon if she was not capable of continuing herself, for example if tiredness was affecting her ability to perform the operation safely. This balance may be difficult, and it was also described by Pia:

*“I also feel a bit that it’s my responsibility to make sure that I don’t work so many hours that I can’t manage in reality to look after my body ( … ) then you have to fight to overcome that pressure that you have to work a lot all the time, and it is a bit so that I can stay comfortable with myself and in good shape.” (Pia)*

It is the surgeons themselves who carry the responsibility of balancing these challenges and making decisions both about the short-term (if they need a break) and about the long term (to improve their physical capacity by doing physical exercise training). In most cases, the surgeons do not tend to give their own health the highest priority. This is evident when they describe their culture as “Tarzan-like”. The surgeons perform operations even if it negatively affects their health:

*“I know plenty of colleagues who stands operating with tearing, burning pain down one leg or who have to take painkillers just to be able to be there at all.” (David)*

Painkillers are used as a quick short-term solution, providing the surgeons with the ability to do their job and take responsibility for the patients.

*“When Mogens comes back looking like a broken number 7, I’m perfectly aware that it’s his back hurting him. It’s not that difficult to work out. Then he just takes a couple of ibuprofens, and we keep rolling, don’t we?” (Christian)*

##### How to handle it

The surgeons have tried a range of methods to manage their musculoskeletal pain. Initiatives initiated by the management e.g. supervision with a person from the occupational clinic, as well as individual strategies e.g. consulting a physiotherapist. What is common is that the surgeons also describe why it has not been successful.

#### Work-related focus on physical ergonomics

One surgeon mentioned that a representative for the clinic of occupational medicine once visited the operation theatre. It was, however, unclear whether this was an initiative from the management or what, and the surgeon felt that the initiative lacked connection to his daily routines:

*“A man came from the clinic for occupational medicine to look at how we stood during the operations, and he had no difficulty in seeing that it was a problem, but apart from that nothing happened ( … ) He just took a look and took pictures to provide documentation of the way we stood and for how long, but it was as though that was the end of it.” (Andreas)*

Some of the surgeons explained that they felt uncertain because they lacked the knowledge on how to adjust the available equipment and their workplace layout as well as other potentially beneficial initiatives.

*“We just don’t know how best to use it ( … ). In reality, we should have someone or other who knows about it who can tell us: This is how the chair should be positioned every time.” (Martin)*

The surgeons were aware of the importance of correct work posture but were insecure how to deal with it. One surgeon described how he had consulted a physiotherapist regarding his own work posture:

*“So I paid out of my own pocket, in fact, to get an occupational trial assessment at a physiotherapist’s, and then we play-acted an operation game, and then she told me off about how I ought to stand, and yes, there probably was a difference between how I stood and how I should have been standing, and then she devised a specially created back exercise programme, which I had to admit did help.” (Christian)*

#### Micro-breaks and physical activity

One surgeon explained that she was aware that there was the opportunity to ease the workload during operations. It was, however, a problem for her to take advantage of it. For example, when something hurt during an operation:

*“If you’re involved in lengthy operations, to make it less physically demanding you can take occasional breaks, and that’s something we really ought to do more ( … ) but people don’t do it ( … ) I don’t think there has been much of a focus on it, I mean the idea of taking breaks, here on this ward – unfortunately.” (Pia)*

The surgeons demonstrated some knowledge of possible solutions. One solution, for example, was to break up their work-load. However, the surgeons did not make use of this opportunity during their work day, and nor did they indicate any collective understanding or regulations for breaks.

At the same time, the surgeons were aware that their physical capacity could increase their work ability and prevent musculoskeletal pain. Some of the surgeons described running or biking in their leisure time to become physically fit as a preparation for their job as surgeon:

*“I can feel that doing some training away from work – that helps! ( … ) you can’t manage without it. Previously I didn’t need to keep in form to prevent neck, shoulder, legs and sciatica and all sorts – it’s started to creep up, so I am forced to keep myself in form, for otherwise I have a bad something or other.” (Martin)*

#### Experiences with health promotion initiatives

The surgeons expressed thoughts and considerations as to different potential health promoting initiatives e.g. exercises with resistance bands:

*“We had a theme day a few years ago, where some people came and showed us some resistance bands we were supposed to stand and pull, some classic stuff for secretaries, and I can’t stand it. I’m sorry. It’s just not for me ( … ) I think it’s ridiculous! Then rather play half an hour of table tennis and have fun doing that.” (David)*

The lack of motivation for activities with resistance bands expressed by this surgeon may be explained by the fact that he had not experienced musculoskeletal pain related to his work. When asked, he reflected that he might be more motivated to take up this kind of preventive initiative if he had musculoskeletal problems.

As described earlier, the surgeons consider musculoskeletal pain a private matter, and one of the surgeons underlined this by rejecting the idea of conducting physical exercise training at work:

*“I mean, I am here for the sake of the patients, and I am here because I am paid to carry out that job. I do not get paid for having to go down and be in a training centre to lift weights, that is not why I am here, that’s something I do in my leisure time.” (Susanne)*

Even though surgeons may be negative about doing physical exercise training during working hours, it is crucial that the management genuinely support and prioritise surgeons’ health.

*“All well and good that management have been given a check - they have now made the health-promoting initiatives - but one pretty important thing is missing, namely that when you get home after a day’s work, that time is actually prioritised in our everyday work for it. And it isn’t, because we’re told 'that’s something you can do when you have time” (Christian)*

Providing the time for surgeons to carry out health promotion initiatives as part of maintaining their work ability is essential for success.

### Logic model of the problem and overall goal

**Fig. 2:**
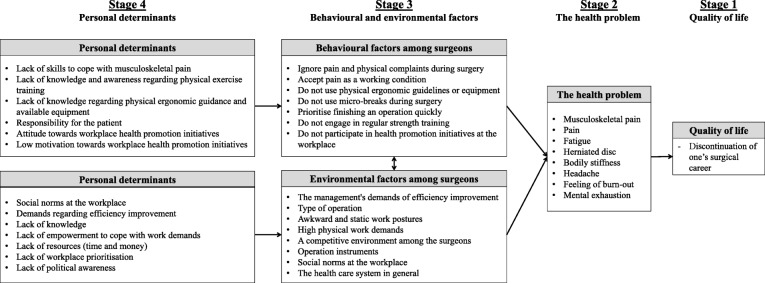
The Logic Model of the Problem

Based on the existing literature and the semi-structured interviews, a needs assessment was conducted to create a logic model of the problem as summarised in Fig. 2. Task 1 identified how musculoskeletal pain will affect the surgeons’ quality of life; for example, if the surgeon was forced to stop his/her surgical career. Task 2 identified the problem and the physical consequences that musculoskeletal pain has for the surgeons, such as bodily stiffness, muscular fatigue and mental exhaustion. Task 3 identified the behavioural and environmental factors that are believed to cause the surgeons’ musculoskeletal pain. The behavioural factors identified were for example accepting pain as a working condition, not using ergonomic equipment, or not engaging in physical exercise training. Environmental factors that may contribute to surgeons’ musculoskeletal pain were the awkward and static work posture, high physical demands, and social norms at the workplace. Finally, in task 4, the personal determinants were identified and separated, based on which factors affected the surgeons’ behaviour or environment. Personal determinants affecting surgeons’ behaviour included lack of knowledge of how to apply the physical ergonomic guidance, lack of skills to cope with musculoskeletal pain, and low motivation towards health enhancing initiatives at the workplace. Personal determinants affecting surgeons’ environment included personal norms adopted from the social environment, lack of resources (time and money), demands regarding efficiency improvement, and lack of political awareness.

The overall goal was based on the high prevalence of musculoskeletal pain identified in the literature search and confirmed in the semi-structured interviews. Thus, the overall goal for the intervention was to identify ways to prevent and rehabilitate musculoskeletal pain among surgeons. This overall goal was subdivided into three categories. Primary prevention, with the aim of avoiding the development of musculoskeletal pain in all surgeons. Secondary prevention, with the aim of tracing and preventing the development of musculoskeletal pain among those surgeons who are in a high-risk group. Tertiary prevention, with the aim of rehabilitating musculoskeletal pain, preventing the recurrence or consequences of having chronic pain.

### Step 2: Specifying performance and change objectives

The logic model of the problem formed the offset for the first task in step two, namely, to formulate health enhancing outcomes for the intervention and to further divide this into a behavioural outcome and an environmental outcome. The behavioural outcome was determined as: *Surgeons should be able to prevent and rehabilitate their musculoskeletal pain*. The environmental outcome was determined as: *The hospital and the management should prioritise surgeons’ work-related musculoskeletal pain and be an active partner in the prevention and rehabilitation process.*

From the two listed outcomes, performance objectives were formulated. The performance objectives describe the actions needed to be achieved by the two defined outcomes.

Four performance objectives were formulated for the behavioural outcome:

1. Surgeons engage in physical exercise strength training to enhance their strength and thereby reduce the relative load on their muscles
2. Surgeons gain knowledge of how their work and awkward and static work postures affect their health.
3. Surgeons gain knowledge of physical ergonomic guidelines and how to use ergonomic equipment
4. Surgeons incorporate micro-breaks during surgery to prevent muscle fatigue

Two performance objectives were formulated for the environmental outcome:

1. The management prioritise surgeons’ work-related musculoskeletal pain
2. The hospitals incorporate strategies and action plans focusing on surgeons’ musculoskeletal pain

To achieve the above performance objectives, we identified six theoretical determinants that were deemed useful in achieving each performance objective and thereby changing the desired outcomes. The six theoretical determinants comprise knowledge, skills, attitude, social influence, self-efficacy, and expected outcomes (Table 2) and originate from Theory of Planned Behavior [35] and Social Cognitive Theory [36]. These two theories both contribute with determinants within behaviour and the surrounding environment’s impact in relation to behavioural change [23].

**Table 2:** Matrix of change objectives created by crossing the performance objectives (behavioural performance objectives 1–4 and environmental performance objectives 5–6) with the theoretical determinants

Performance objectives	Theoretical determinants					
	Knowledge	Skills	Attitudes	Social influence	Self-efficacy	Outcome expectations
1. *Surgeons* perform strength training to reduce the relative load on muscles	Have knowledge about strength training effects and how to perform training	Possess the skills to perform strength training	Acknowledge the meaning of strength training as health promoting	Management and surgeons give priority to strength training and surgeons support each other	Feel confident with performing strength training and believe in own abilities	Expectation that strength training prevents musculoskeletal pain
2. *Surgeons* gain knowledge on how their work impacts their health	Have knowledge about how operations can impact on the musculoskeletal system	Possess the skills to prevent negative physical influence of work	Accept that musculoskeletal pain may be a consequence of being a surgeon	Management and surgeons build a common understanding of potential negative impacts on health	Believe they are capable of preventing work-related musculoskeletal pain	Expectation that prevention of musculoskeletal pain will lead to a healthier working life
3. *Surgeons* learn to use physical ergonomic guidelines and available equipment	Have knowledge about physical ergonomic guidelines and how to use available equipment	Possess the skills to use physical ergonomics and available equipment	Accept and are positive about the use of physical ergonomics	Management and surgeons support colleagues’ use of ergonomic equipment	Feel confident with using ergonomic equipment and believe in own abilities	Expectation that physical ergonomics has a preventive effect on musculoskeletal pain
4. *Surgeons* incorporate micro-breaks during surgery to prevent muscle fatigue	Have knowledge about how to use micro-breaks	Possess the skills to implement micro-breaks during operations	Accept and are positive about the usefulness of micro-breaks during operations	Management and surgeons support colleagues’ use of micro-breaks.	Believe in their ability to implement micro-breaks	Expectation that use of micro-breaks has a preventive effect on musculoskeletal pain
5. *Management* prioritise surgeons’ health	Have knowledge about surgeons’ work-related challenges and impact on musculoskeletal health	Possesses the skills for continuously prioritising surgeons’ musculoskeletal health	Make clear the meaningfulness and importance of giving priority to preventive and rehabilitating strategies	Support relevant strategies e.g. physical ergonomics and physical exercise training	Believes that strategies for prevention of musculoskeletal pain are feasible and effective	Expectation that if the management actively prioritises prevention of musculoskeletal pain among surgeons, the surgeons will also prioritise the issue themselves.
6. The *hospital* incorporates strategies and action plans focusing on surgeons’ musculoskeletal pain	Has knowledge about prevention and rehabilitation of work-related musculoskeletal pain	Possesses the skills to continuously perform and renew strategies for prevention and rehabilitation of musculoskeletal pain	Makes clear the meaningfulness and importance of surgeons’ musculoskeletal health	Provides support to strategies that allow surgeons to perform their job without experiencing work-related musculoskeletal pain	Believes that strategies and action plans can prevent and rehabilitate musculoskeletal pain among surgeons	Expectation that by having strategies and action plans ready for the surgeons, the surgeons feel more obliged to engage, which is likely to improve efficiency

Knowledge is often considered as a precondition for other determinants. In Theory of Planned Behavior and Social Cognitive Theory, knowledge per se does not create a change but is essential for the other determinants to create a behavioural change. From the literature search and the semi-structured interviews, it was evident that surgeons lack knowledge about physical ergonomics in the operation theatre and that the incorporation of micro-breaks may relieve their pain. In addition, enhancing surgeons’ knowledge regarding the benefits of physical exercise training, may facilitate a change or influence the other determinants.

In relation to achieving a higher level of knowledge, the development of new skills or competences is crucial to facilitate the desired change. Many of the surgeons claimed that they had not received any specific training on proper work postures to lower the physical demands or how to use ergonomic equipment in the operation theatre. Likewise, they indicated that the management did not support the surgeons with education, knowledge-sharing or was an active participant in this process.

Attitude from Theory of Planned Behavior is very important, as our attitude towards change is pivotal for them to happen. Several of the surgeons demonstrated a negative attitude towards engaging in physical exercise training during work hours. Work hours should not be used to ensure surgeons’ own health since patients’ health takes priority. However, surgeons highlighted the importance of being physically active, and many of the surgeons engaged in physical endurance exercise training during leisure time.

Social influence deals with the surroundings and refers to the environmental influence on the outcome. During the interviews, several of the surgeons mentioned previous health promotion initiatives at the workplace, but also indicated that management did not seem to appreciate them taking part. They were told to do it when they felt they had the time. This lack of support may hinder potential change.

Self-efficacy from Social Cognitive Theory is equivalent to behavioural control from Theory of Planned Behavior and deals with an individual’s believe in their own competence to change. The likelihood for a given change to happen is greater if the individual believes they are capable of changing their behaviour. From the interviews it was evident that several of the surgeons had taken steps to reduce their pain. They had turned to advice from physiotherapists, for example with regard to work posture or exercises to relieve pain. Likewise, a study demonstrated that 85% of the surgeons had received treatment for their work-related pain [4]. Thus, several of the surgeons demonstrated a high degree of behavioural control.

Expected outcomes relate to the individual’s positive or negative views of the potential consequences of a given change [36]. Some surgeons indicated that they were not motivated to participate in resistance band strength training exercises at the workplace. Such scepticism may seem obstructive during an attempt to implement health promoting actions; e.g. physical exercise training for the surgeons. However, you may be able to facilitate participation, if you manage to convince the surgeons that preventive physical exercise training or micro-breaks would be beneficial for their work-related musculoskeletal pain, for their future as surgeons, and, above all, for the health of patients.

**Fig. 3:**
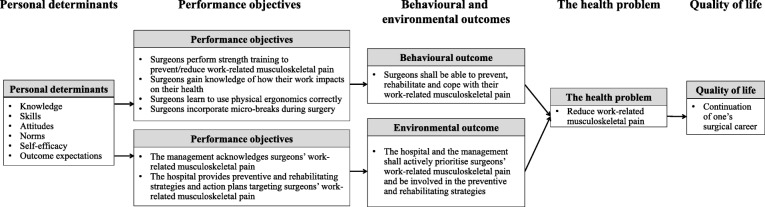
The Logic Model of the Change

The last task in step two was to set up the logic model of change. This model contains knowledge from the preceding steps and illustrates the pathways of an effective intervention (Fig. 3).

### Step 3: Theory-based and practical strategies for prevention of musculoskeletal pain

To identify practical strategies for the interventions, the six theoretical determinants and change objectives were matched with theoretical methods and strategies in a matrix (Table 3).

**Table 3:** Theoretical determinants, methods and practical strategies

Theoretical determinants Change objectives	Theory-based methods	Practical strategies
Knowledge The benefits of strength training Impact of work on musculoskeletal health Physical ergonomic guidelines and ergonomic equipment The benefits of micro-breaks	Personalise risk Modelling Active learning	Mandatory workshops must be introduced on surgical wards to give surgeons knowledge about potential musculoskeletal pain while working as a surgeon, and about the prevention and rehabilitation of musculoskeletal pain
Skills Perform strength training Prevent negative impacts of work Use ergonomic equipment Perform micro-breaks	Guided practice Active learning	At workshops surgeons learn skills to prevent and rehabilitate musculoskeletal pain
Attitude Prioritise physical exercise training Acceptance of negative impact of work on musculoskeletal health Positive acceptance of ergonomic equipment Acceptance of usefulness of micro-breaks Articulation and shared understanding of working conditions	Modelling Verbal persuasion	Appointed health ambassadors help produce a positive attitude to health promotion initiatives on local surgical wards
Social influence Surgeons, management, and the hospital build up a positive and sustainable healthy working environment on surgical wards	Verbal persuasion Modelling	Surgeons and management prioritise and enforce the importance of health promotion initiatives. Ward management initiates upstart meetings with newly appointed surgeons to promote health initiatives.
Self-efficacy Believe in their ability to organise exercise training Believe that a preventive effort will pay off in the long-term Believes in the preventive effect of ergonomic equipment Believe in the preventive effect of micro-breaks	Modelling Active learning Verbal persuasion	Appointed health ambassadors initiate and maintain health promotion initiatives on local surgical wards. Ward management establish the structures that make it possible for surgeons to perform health promotion activities at work.
Outcome expectations Strength training leads to a healthier musculoskeletal system Prioritising micro-breaks leads to a healthier musculoskeletal system Using ergonomic equipment leads to a healthier musculoskeletal system Mutual understanding of work-related musculoskeletal pain leads to acceptance and better musculoskeletal health	Modelling Verbal persuasion	At workshops, surgeons receive knowledge about the consequences of suffering from musculoskeletal pain At workshops surgeons get detailed knowledge about benefits from health promotion activities. Health ambassadors support surgeons’ health and initiatives for maintaining good work ability for surgeons.

From the Social Cognitive Theory, the theoretical methods of modelling and verbal persuasion were applied to change the determinants attitude, social influence, self-efficacy and expected outcomes [28]. Similarly, active learning and guidance from Social Cognitive Theory were applied to affect the determinants’ skills and self-efficacy. In addition, the method “Personal risk” from the Precaution – Adoption Process Model was applied to change the determinants’ knowledge and expected outcomes [28]. Using “Personal risk” as a method may provide motivation and give the surgeons knowledge about the personal consequences that are associated with their current behaviour. It may promote them to act because of the new knowledge and a positive mind-set towards expected outcomes.

Modelling and verbal convincing were applied for the surgeons to see that others have succeeded with the same behaviour. Appointing role models or health ambassadors that facilitate the intervention and success with it may prompt the rest of the group to follow, because it enhances their self-efficacy towards the new behaviour. It is important to involve the management at this stage, as an active and visible management may promote the surgeons to take part and thereby facilitate new social influence.

Active learning and guidance were applied to give the surgeons the competences and skills to accomplish the intervention, for example, by demonstrating physical ergonomic adjustments in the operation theatre or how to perform physical exercises to relieve pain. It is important that the surgeons become familiar with the new strategies and believe they have the competences and skills to execute the practical strategies correctly.

Table 4 presents the intervention design plan, summarising the focus areas identified in step 2 as well as the relevant strategies suggested in step 3.

**Table 4:** Intervention strategies targeting surgeons’ work-related musculoskeletal pain

Strategies	Content	Target group
Physical exercise training	Mandatory workshops with knowledge-sharing on wards Topic: “Do your physical exercise training – It matters for your health” Physical exercise training Individualised physical exercises targeting vulnerable and painful body regions as well as other health risk indicators	The surgeons
Physical ergonomics	Mandatory workshops with knowledge-sharing on wards Topic: “Use available physical ergonomics and sustain your work ability*”* Guided training Training and counselling in available physical ergonomics tailored to each surgeon	The surgeons
Micro-breaks	Mandatory workshops with knowledge-sharing on wards Topic: “How to incorporate micro-breaks effectively*”* Guided training Training in micro-breaks during surgery	The surgeons
Knowledge of how surgeons’ work impacts on surgeons’ health	Mandatory workshops with knowledge-sharing on wards Topic: “My work impacts on my health – what can I do?” Guided training Sessions on how to cope with musculoskeletal pain (e.g. 3 conversations with a professional).	The surgeons
The hospital and the management prioritise surgeons’ work-related musculoskeletal pain	Mandatory workshops with knowledge-sharing on wards Topic: “Prioritising surgeons’ work-related musculoskeletal pain” Guided training The management and the hospital receive advice from an external consultancy on how to implement and prioritise the above strategies.	The management and the hospital

## Discussion

The aim of this paper was to describe the systematic development of feasible strategies that can prevent and rehabilitate surgeons’ musculoskeletal pain, by using an Intervention Mapping approach. Intervention Mapping proved to be a helpful tool in pinpointing the needs of the surgeons while developing practical strategies that can be applied for this occupational group.

We conducted a needs assessment, examined the existing literature and interviewed six surgeons. From this, it was evident that musculoskeletal pain is an issue that needs attention, as it may influence on the surgeon’s future career. It was also evident that surgeons do not talk about their complaints, and that they have developed a certain culture – ‘*if you cannot stand the pace, then you are out*’. Additionally, most surgeons were not aware of opportunities to lower the physical demands in the operating theatre, e.g. how to vary work posture and how to place the surgical equipment to avoid awkward work postures etc. The results also showed that to succeed with prevention and rehabilitation of musculoskeletal pain among surgeons, it is important to involve the management and have their full support. The management’s prioritisation is crucial – especially regarding scheduling and allowing the time for surgeons to engage in health promotion initiatives.

Theoretical determinants from Theory of Planned Behavior and Social Cognitive Theory were applied in the process of determining why and how to change behaviour. Altogether, this proceeded to the development of five strategies targeting physical exercise training, physical ergonomics, micro-breaks, knowledge of how surgeons’ work impacts on surgeons’ health, and how can the hospital and management prioritise surgeon’s work-related musculoskeletal pain. The Intervention Mapping approach was chosen as surgeons represent a complex occupational group, and to overcome what has previously been shown to be one of the big challenges of workplace interventions; limited effect due to substantial drop-out and low adherence to the intervention in question [37–40]. Within the past decade, the use of Intervention Mapping has increased, and it is now acknowledged as a useful, systematic and practical framework in the development of intervention designs [26, 27, 41].

Surgeons’ work is multifunctional and includes a number of different tasks, long shifts, and a work schedule that can be unpredictable. Thus, it may be hard to implement a successful intervention without a participatory approach. It should also be acknowledged that the optimal approach can vary from hospital to hospital or even from ward to ward. For instance, it was interesting to witness from the interviews that an initiative regarding physical exercise training within work hours may not be optimal for this occupational group. However, the surgeons were aware of the importance of being physically fit, thus, such initiative could be offered outside work hours. Also interesting was the value of a visible and active management, which according to the surgeons could be optimised. To succeed, the authors underline the importance of involving the management and identifying potential facilitators and barriers.

### Strengths and limitations of the study

The systematic approach used in Intervention Mapping is a strong feature, as the experience and information obtained in the process of tailoring and developing the intervention will be captured and, hopefully, benefit the present as well as future studies. A limitation in the present study is the low number of interviewed surgeons. Although the literature supported the essence of the interviews, we do not know if the results are generalisable in relation to implementation in other hospitals. There may be differences between hospitals and even specialties and it is important to use a participatory approach for the next three steps in the Intervention Mapping approach. The authors believe the proposed strategies are worth carrying out, possibly with small local adjustments.

## Conclusion

The present Intervention Mapping study demonstrated that musculoskeletal pain among surgeons is a complex area that needs attention. Our findings highlight a need for behavioural changes of the surgeons as well as organisational and attitudinal changes for the management. Future research could focus on the design and evaluation of the effect of focused interventions.
